# Host Genetic Factors and Dendritic Cell Responses Associated with the Outcome of Interferon/Ribavirin Treatment in HIV-1/HCV Co-Infected Individuals

**DOI:** 10.4172/2155-9899.1000271

**Published:** 2014-10-31

**Authors:** Mohit Sehgal, Marija Zeremski, Andrew H. Talal, Zafar K. Khan, Renold Capocasale, Ramila Philip, Pooja Jain

**Affiliations:** 1 Department of Microbiology and Immunology, Drexel University College of Medicine, Philadelphia, PA, USA; 2 School of Medicine and Biomedical Sciences, University at Buffalo, Buffalo, NY, USA; 3 Flowmetric, Inc., Pennsylvania Biotechnology Center, Doylestown, PA, USA; 4 Immunotope, Inc., Pennsylvania Biotechnology Center, Doylestown, PA, USA

**Keywords:** Dendritic cells, Interferon-α, Ribavirin, human chronic viral infections, HIV-1, HCV, HIV-1/HCV co-infection

## Abstract

HIV-1/HCV co-infection is a significant health problem. Highly active antiretroviral treatment (HAART) against HIV-1 has proved to be fairly successful. On the other hand, direct acting antiviral drugs against HCV have improved cure rates but high cost and development of drug resistance are important concerns. Therefore PEGylated interferon (PEG-IFN) and ribavirin (RBV) still remain essential components of HCV treatment, and identification of host factors that predict IFN/RBV treatment response is necessary for effective clinical management of HCV infection. Impaired dendritic cell (DC) and T cell responses are associated with HCV persistence. It has been shown that IFN/RBV treatment enhances HCV-specific T cell functions and it is likely that functional restoration of DCs is the underlying cause. To test this hypothesis, we utilized an antibody cocktail (consisting of DC maturation, adhesion and other surface markers) to perform comprehensive phenotypic characterization of myeloid DCs (mDCs) and plasmacytoid DCs (pDCs) in a cohort of HIV-1/HCV co-infected individuals undergoing IFN/RBV treatment. Our results show that pre-treatment frequencies of mDCs are lower in non-responders (NRs) compared to responders (SVRs) and healthy controls. Although, the treatment was able to restore the frequency of mDCs in NRs, it downregulated the frequency of CCR7^+^, CD54^+^ and CD62L^+^ mDCs. Pre-treatment frequencies of pDCs were lower in NRs and decreased further upon treatment. Compared to SVRs, NRs exhibited higher ratio of PD-L1^+^/CD86^+^ pDCs prior to treatment; and this ratio remained high even after treatment. These findings demonstrate that enumeration and phenotypic assessment of DCs before/during therapy can help predict the treatment outcome. We also show that before treatment, PBMCs from SVRs secrete higher amounts of IFN-γ compared to controls and NRs. Upon genotyping *IFNL3* polymorphisms rs12979860, rs4803217 and ss469415590, we found rs12979860 to be a better predictor of treatment outcome. Collectively, our study led to identification of important correlates of IFN/RBV treatment response in HIV-1/HCV co-infected individuals.

## Introduction

Chronic infections with human immunodeficiency virus 1 (HIV-1) and hepatitis C virus (HCV) are a leading cause of morbidity and mortality worldwide. Chronic infection with HIV-1 causes acquired immunodeficiency syndrome [1], which is characterized by severe immunosuppression and associated opportunistic infections, whereas chronic HCV infection causes chronic liver disease and its associated complications such as liver cirrhosis, liver failure and hepatocellular carcinoma [2]. HCV genotype 1 is the most prevalent and difficult to treat genotype [3]. Up to 30% of HIV-infected individuals overall and 60–90% of HIV-infected injection drug users are infected with HCV [4–6] and therefore HIV-1/HCV co-infection is a significant health problem. Since co-infection with HIV-1 is known to have a negative impact on HCV pathogenesis and disease progression, it is recommended that in individuals with HIV-1/HCV co-infection, HIV-1 be controlled prior to initiation of HCV treatment [7]. PEG-Interferon-α (PEG-IFN)/Ribavirin (RBV) combination therapy has been the mainstay of chronic HCV treatment but unlike HAART it has been difficult because of limited success rate (~50%) and toxic side effects. Both PEG-IFN and RBV have nonspecific and largely unknown mechanisms of action and therefore in the last few years direct-acting antiviral agents (DAAs) have been developed and approved for treating HCV. Administration of DAAs with/without IFN/RBV has improved cure rates but the high cost of DAAs along with emergence of drug-resistant HCV variants remain important concerns. Therefore, for better clinical management of HCV, it is necessary to identify host factors that predict the outcome of IFN/RBV treatment. Individuals with undetectable HCV RNA levels six months or longer after IFN/RBV treatment are referred to as sustained virological responders (SVRs) and those that fail to clear HCV after treatment are referred to as non-responders (NRs). As sentinels of our immune system, DCs play a central role in initiating and regulating protective antiviral immunity. Immature DCs express inflammatory chemokine receptor CCR5 whose interaction with chemokine CCL5 promotes their recruitment to the site of infection. At the site of infection, DCs sense viral signatures through pattern recognition receptors (PRRs) and become activated. Upon activation, DCs undergo a process of maturation which involves increase in the surface expression of major histocompatibility complex (MHC) class I and II molecules, co-stimulatory molecules (such as CD86), adhesion molecules (such as CD54 and CD62L) and lymph node homing receptors (such as CCR7) [8]. The cellular immune response has a profound impact on outcome and pathogenesis of HCV infection [9]. Poor CD4^+^ and CD8^+^ T cell response is a hallmark of chronic HCV infection [10]. Being professional antigen presenting cells, DCs play a crucial role in directing an efficient antiviral T-cell response. Functional defects in DCs have been shown to underlie weak and narrowly focused T cell response characteristic of chronic HCV infection [11,12]. Compared to uninfected individuals, DCs isolated from HCV-infected individuals are less efficient in stimulating T cell activation and proliferation [12–14]. HCV-infected individuals have increased expression of programmed death-ligand 1 (PD-L1) on DCs [15,16]. Binding of PD-L1 to programmed cell death protein 1 (PD-1) expressed on T cells delivers a coinhibitory signal to T cells resulting in their reduced proliferation and effector functions. Also, HIV-1-infected [17–21] and HCV-infected [12,22–24] individuals have reduced frequencies of mDCs and pDCs compared to uninfected individuals. Since IFN/RBV therapy is known to enhance HCV-specific CD4 and CD8 T-cell response in SVRs, it is possible that IFN/RBV therapy restores both DC frequencies and functions in SVRs but not in NRs. Most studies that have investigated the role of DCs in IFN/RBV treatment outcome in HCV mono-infected or HIV-1/HCV co-infected individuals have mostly focused on HCV genotype 1 since it is the most prevalent and difficult to treat HCV genotype. Herein, we investigated a cohort of HIV-1/HCV (HCV genotype 1) co-infected individuals to study whether IFN/RBV treatment differentially affects DC frequencies and phenotype in NRs and SVRs. We also genotyped three important SNPs associated with treatment response. Collectively, this study helped identify the genetic and immune correlates of IFN/RBV treatment response in HIV-1/HCV individuals.

## Results

### Impact of SNPs rs12979860, rs4803217 and ss469415590 on the outcome of PEG-IFN/RBV treatment in HIV-1/HCV co-infected individuals

**Figure 1:**
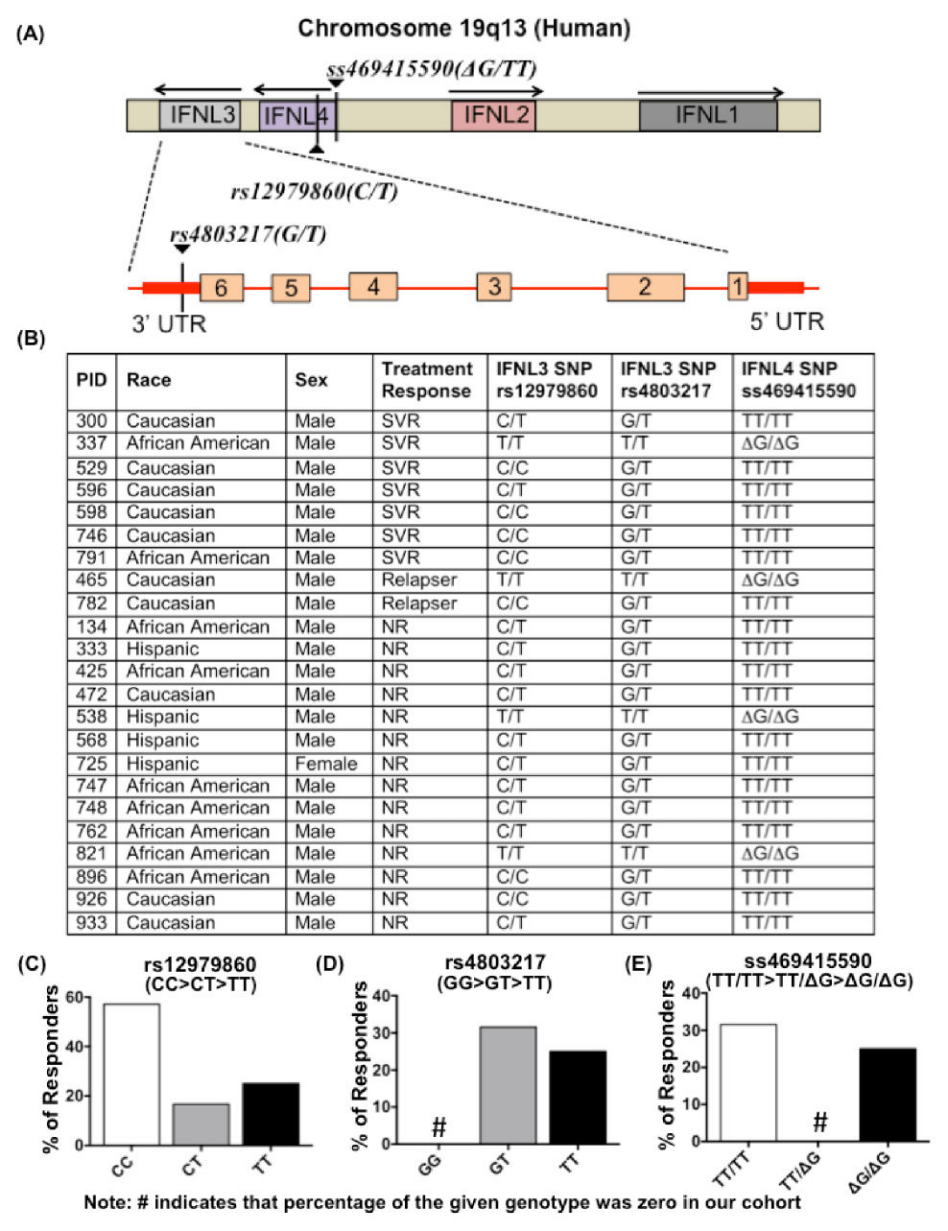
Impact of SNPs rs12979860, rs4803217 and ss469415590 on the outcome of PEG-IFN/RBV treatment in HIV-1/HCV co-infected individuals (A) Schematic representing the location of IFNL1-4 on chromosome 19. SNPs rs12979860 and ss469415590 are located upstream of IFNL3 whereas SNP rs4803217 is harbored in the 3′ UTR of IFNL3. (B) Table showing the race, sex, treatment response as well as rs12979860, rs4803217 and ss469415590 genotypes (determined using custom designed TaqMan qPCR based allelic discrimination assay, Life Technologies) in a cohort of HIV-1/HCV co-infected individuals utilized in the study. (C) Bar graph indicating the percentage of sustained virological responders in individuals carrying the given rs12979860 genotype (CC/CT/ TT). (D) Bar graph indicating the percentage of responders in individuals carrying the given rs4803217 genotype (GG/GT/TT). (E) Bar graph indicating the percentage of responders in individuals carrying the given ss469415590 genotype (TT/ΔGT/ ΔGΔG).

Genome-wide association studies have identified a single nucleotide polymorphism (SNP) rs12979860 (CC>CT>TT) which is strongly associated with treatment-induced as well as spontaneous clearance of HCV in HCV mono-infected as well as HIV-1/HCV co-infected individuals [25]. It is located upstream of IFNL3 (which encodes IFN-λ3, also known as interleukin 28B). However, the molecular mechanisms underlying this association have remained unclear. Recently, two functional SNPs rs4803217 and ss469415590 that are in linkage disequilibrium with rs12979860 have received considerable attention (All three SNPs are schematically represented in Figure 1A). SNP rs4803217 (GG>GT>TT) located in 3′ UTR of IFNL3 has been shown to affect the outcome of HCV infection (by influencing AU-rich element-mediated decay of IFNL3 mRNA) as well as response to IFN/RBV treatment [26–28]. SNP ss469415590 (TT/TT>ΔG/TT>ΔG/ ΔG) located upstream of IFNL3 has also been shown to affect spontaneous as well as treatment-induced clearance of HCV genotypes 1 and 4 (more difficult to treat genotypes compared to genotypes 2 and 3) [29–31]. The unfavorable allele (represented as ΔG) is characterized by deletion of a single base, which causes a frameshift mutation leading to expression of a novel protein IFNL4. It is suggested that IFNL4 induces a set of unfavorable genes that allow increased viral replication and therefore its absence from HCV-infected cells has a protective effect against HCV. The role of rs4803217 and ss469415590 in IFN/RBV treatment-induced HCV clearance is not extensively studied in HIV-1/HCV co-infected individuals. In order to understand whether these three SNPs can influence the outcome of IFN/RBV treatment by affecting DC functions, it is important to first validate that they are indeed capable of predicting the treatment response in our cohort. Therefore, we investigated the association between these three SNPs and IFN/RBV treatment response in a cohort of HIV-1/HCV co-infected individuals.

Figure 1B shows the gender, race, treatment response, and genotypes of all three SNPs. As expected, rs12979860 showed a strong association with treatment response with ~60% of individuals with the most favorable genotype CC and only 25% of individuals with the least favorable genotype TT achieving sustained virological response (SVR) (Figure 1C).

In our cohort, no individual possessed the most favorable rs4803217 genotype (GG) (Figure 1D). Approximately 33% of the individuals with intermediate genotype GT and only 25% of the individuals with least favorable genotype TT cleared the virus (Figure 1D). Approximately 33% of the individuals with most favorable ss469415590 genotype TT/TT and only 25% of the individuals with least favorable genotype ΔG/ΔG cleared the virus (Figure 1E). Overall, in our cohort rs12979860 proved to be a much stronger predictor compared to rs4803217 and ss469415590. As discussed above, studies with large sample size have shown that SNPs rs4803217 and ss469415590 are strongly associated with SVR. Therefore, in our study, weak association of rs4803217 and ss469415590 with SVR could be due to small sample size. Also, we did not detect any significant association between these three SNPs and DC phenotype (Data not shown). This is not entirely surprising since another study with HIV/HCV co-infected patients recently reported that rs12979860 is not associated with DC activation state. In future, it will still be very useful to develop a comprehensive understanding of the impact of these SNPs on DC functions.

### Effect of IFN/RBV treatment on the frequency of mDCs and pDCs

**Figure 2:**
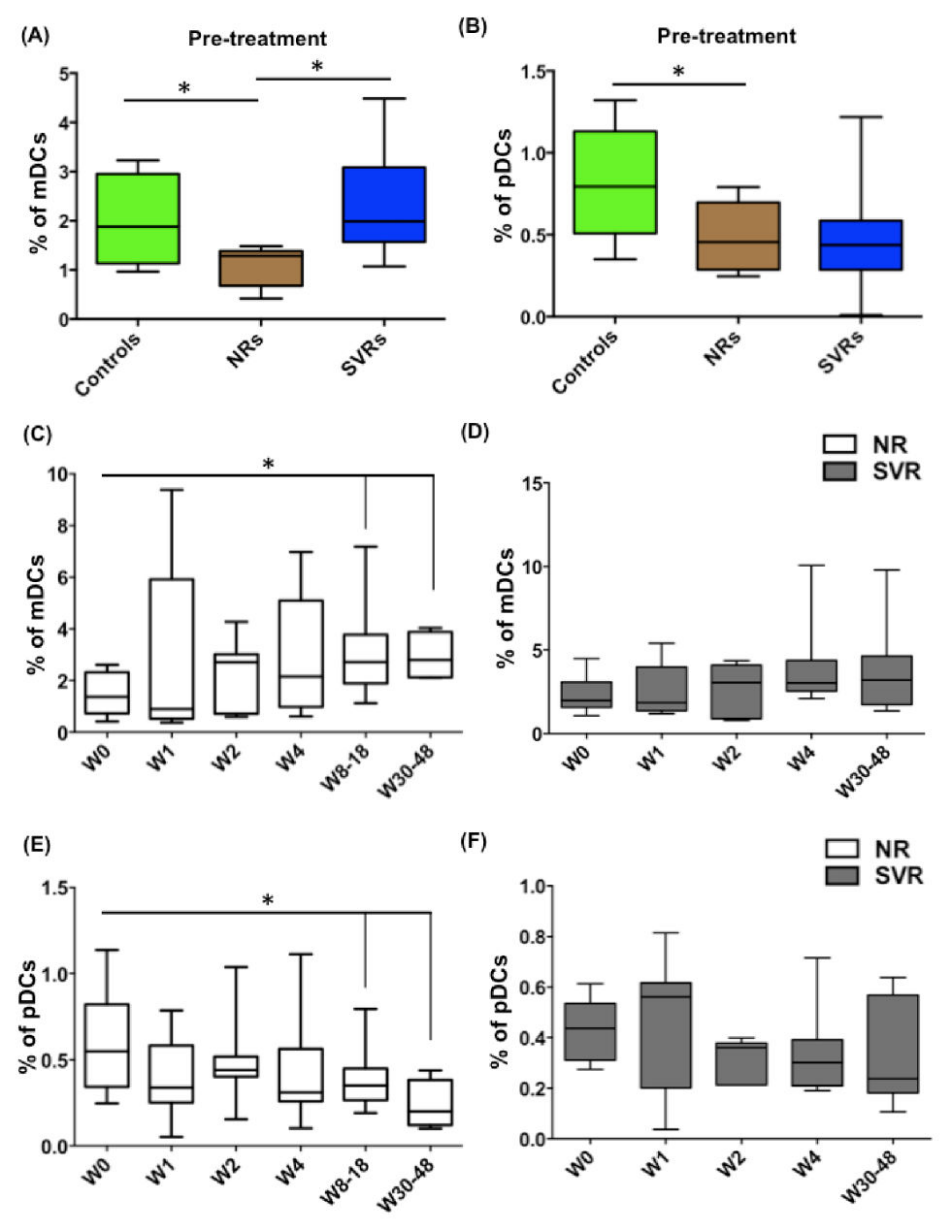
Effect of IFN/RBV treatment on the frequency of mDCs and pDCs in NRs and SVRs PBMCs isolated from seronegative controls, NRs and SVRs were stained with polychromatic antibody cocktail described in supplementary Figure 1. (A) Box and whiskers graph indicating the percentage of mDCs, and (B) pDCs in seronegative controls, SVRs and NRs prior to initiation of treatment. (C) Box and whiskers graph indicating the percentage of mDCs in NRs, and (D) SVRs at different time points during the course of treatment. (E) Box and whiskers graph indicating the percentage of pDCs in NRs, and (F) SVRs at different time points during the course of treatment. P values were calculated using Student’s t test (* represents P<0.05).

Various studies have shown that HIV-1- and HCV-infected individuals have reduced frequency of mDCs and pDCs in blood; however the impact of HIV-1/HCV co-infection on DC frequency and functions is not completely understood. In our study we used flow cytometry based thirteen-color antibody cocktail (details are mentioned in supplementary Figure 1A) for enumeration and phenotypic assessment of two subsets of blood DCs: mDCs (CD11c^+^/ CD123^−^) and pDCs (CD11c^−^/CD123^+^). We observed that prior to initiation of IFN/RBV treatment; NRs had significantly lower frequency of mDCs compared to SVRs and seronegative controls (Figure 2A). Another study reported reduced frequency of mDCs in chronic hepatitis C individuals but interestingly the reduction in frequency was more pronounced in individuals carrying HCV genotype 2 compared to genotype 1 and 3 suggesting that HCV genotype could differentially affect DC frequency possibly due to differences in tropism of each HCV genotype [32]. Frequency of pDCs in NRs was significantly lower compared to controls but not compared to SVRs (Figure 2B). Next, we investigated whether IFN/RBV treatment restores the frequency of mDCs and pDCs in NRs. We found that IFN/RBV treatment led to an increase in the frequency of mDCs in NRs (Figure 2C) but the frequency of pDCs decreased further (Figure 2E). On the other hand, frequencies of mDCs and pDCs remained unchanged in SVRs (Figures 2D and 2F). Opposite to our study, a study with HCV-monoinfected individuals reports that IFN/RBV treatment leads to increase in the percentage of BDCA-1^+^ mDCs in SVRs [33]. However, this could be due to HIV-1 co-infection in our cohort of HCV infected individuals.

### IFN/RBV treatment downregulates the percentage of mDCs expressing CCR7 in NRs

**Figure 3:**
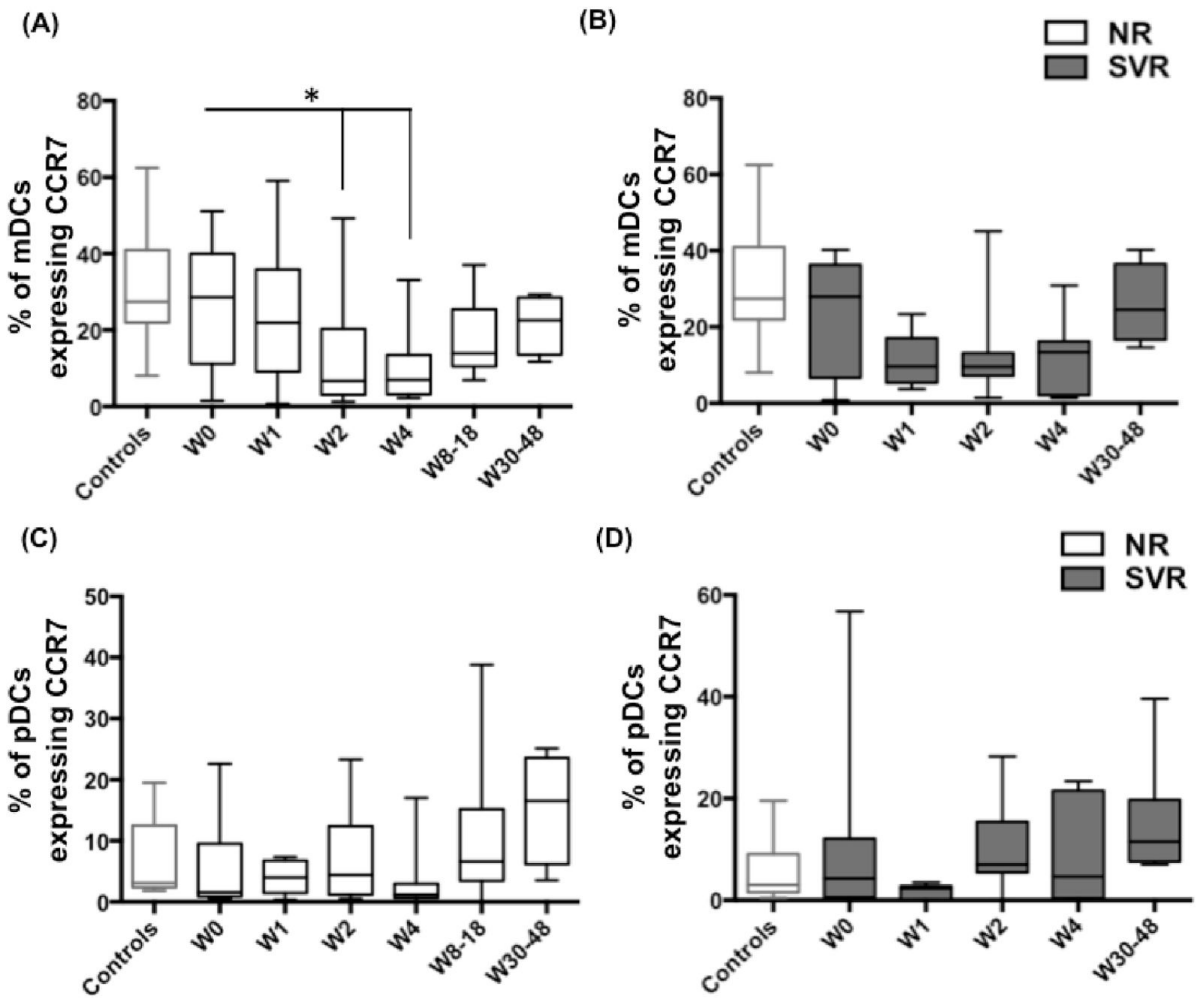
IFN/RBV treatment downregulates the percentage of mDCs expressing CCR7 in NRs PBMCs isolated from seronegative controls, NRs and SVRs were stained with polychromatic antibody cocktail described in supplementary Figure 1. (A) Box and whiskers graph indicating the percentage of mDCs expressing CCR7 in NRs, and (B) SVRs prior to and during the course of IFN/RBV treatment. (C) Box and whiskers graph indicating the percentage of pDCs expressing CCR7 in NRs, and (D) SVRs prior to and during the course of IFN/RBV treatment. P values were calculated using Student’s t test (* represents P<0.05).

In addition to direct antiviral effects, IFN-α plays an important role in shaping the immune response by regulating the maturation, migratory potential and immunostimulatory capacity of DCs. Upon maturation, DCs upregulate CCR7 expression. Failure to upregulate CCR7 inhibits the migration of DCs towards CCL21, a CCR7 binding chemokine that is important for homing of DCs to lymph nodes. We hypothesized that even though IFN/RBV treatment led to restoration of mDC frequency in NRs, the potential of mDCs to migrate to lymph nodes and activate T cells could still be impaired. In this regard, we found that upon treatment, the percentage of mDCs expressing CCR7 was reduced in NRs (Figure 3A) but not in SVRs (Figure 3B). At the end of treatment, the frequency of CCR7^+^ mDCs was lower (although statistical significance did not reach 95% confidence interval) in NRs compared to controls. On the other hand, percentage of pDCs expressing CCR7 remained unchanged in both NRs (Figure 3C) and SVRs (Figure 3D). This shows that diminished frequency of CCR7^+^ mDCs during the first four weeks of IFN/RBV treatment could be a key event in treatment failure.

### Effect of IFN/RBV treatment on DC adhesion markers CD54 and CD62L

**Figure 4:**
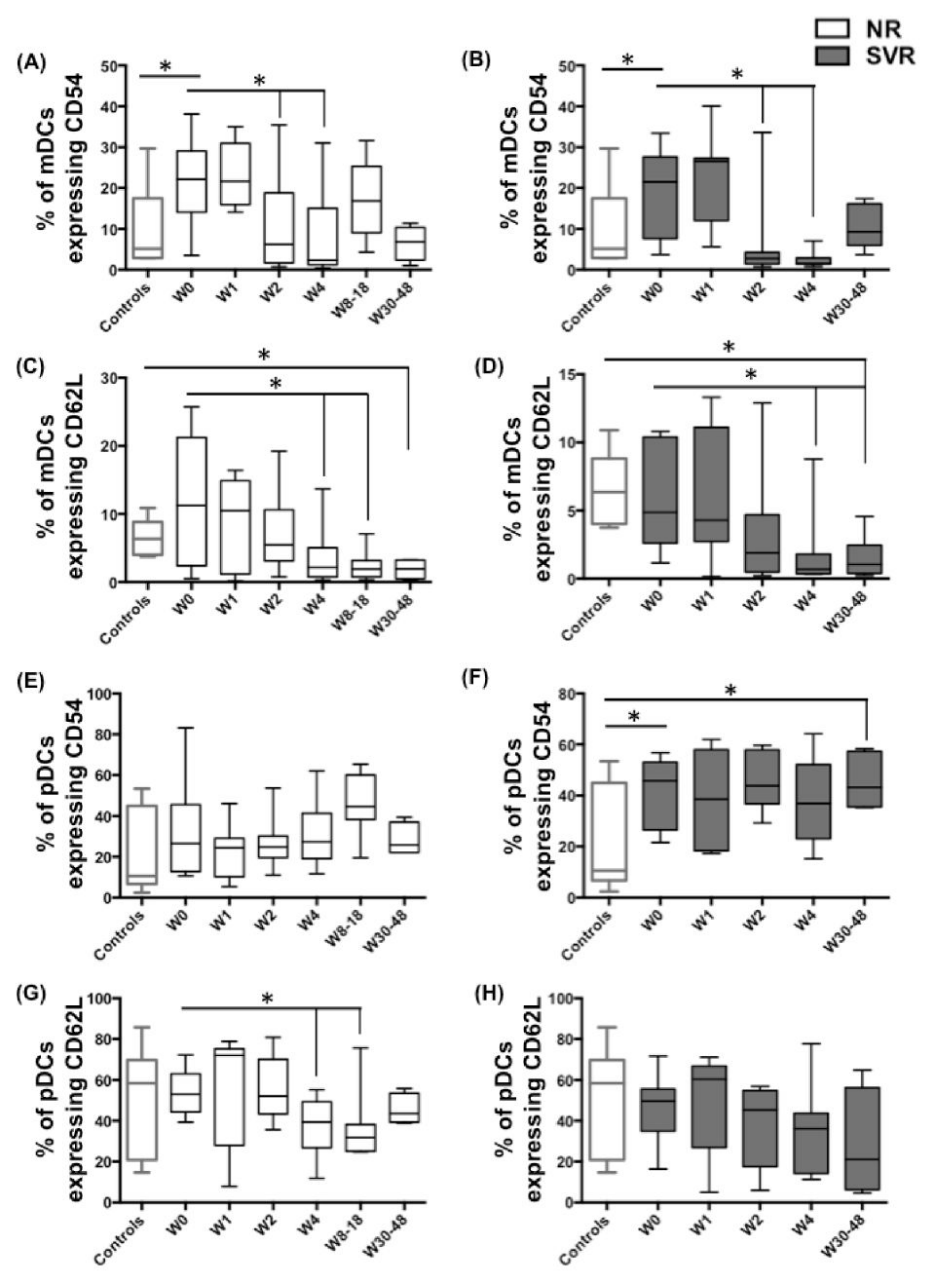
IFN/RBV treatment downregulates the frequency of CD54+ mDCs, CD62L+ mDCs and CD62L+ pDCs in NRs PBMCs isolated from seronegative controls, NRs and SVRs were stained with polychromatic antibody cocktail described in supplementary Figure 1. (A) Box and whiskers graph indicating the percentage of mDCs expressing CD54 in NRs, and (B) SVRs. (C) Box and whiskers graph indicating the percentage of mDCs expressing CD62L in NRs, and (D) SVRs. (E) Box and whiskers graph indicating the percentage of pDCs expressing CD54 in NRs, and (F) SVRs. (G) Box and whiskers graph indicating the percentage of pDCs expressing CD62L in NRs, and (H) SVRs. P values were calculated using Student’s t test (* represents P<0.05).

IFN-α is known to upregulate the adhesion molecules CD54 and CD62L on DCs. Both CD54 and CD62L play an important role in DC-T cell interaction that results in activation of T cells. We investigated the percentage of DCs expressing CD54 and CD62L in NRs and SVRs both before and during the course of treatment. We found that prior to treatment, both NRs (Figure 4A) and SVRs (Figure 4B) had significantly higher frequency of CD54^+^ mDCs compared to controls. On the other hand, frequencies of CD62L^+^ mDCs prior to treatment were similar in controls and infected individuals (Figures 4C and 4D). Upon initiation of IFN/RBV treatment, the frequency of CD54^+^ and CD62L^+^ mDCs dropped in both NRs and SVRs (Figures 4A–D). Interestingly, the post-treatment frequencies of CD62L^+^ mDCs were significantly lower than controls. This suggests that IFN/RBV may have a long-term adverse effect on DC functions in both NRs and SVRs. SVRs had higher pre-treatment frequency of CD54^+^ mDCs and it remained higher throughout the course of treatment (Figure 4F). Similar to mDCs, pre-treatment frequencies of CD62L^+^ pDCs were similar in controls and infected individuals, but upon treatment it dropped significantly in NRs.

### Increased PD-L1/CD86 ratio on pDCs of NRs before and after IFN/RBV treatment

HIV-1/HCV co-infection is characterized by poor, narrowly focused and exhausted HCV-specific T cell response. Our group and others have shown that persistent viruses such as HTLV-1, HIV-1 and HCV can cause T cell exhaustion by upregulating the expression of PD-1 (on T cells) and PD-L1 (on DCs). Binding of PD-L1 and PD-1 delivers a coinhibitory signal to T cells leading to reduced proliferation and effector functions. Since both PD-L1 and CD86 belong to the B7 family, it is possible that the balance between coinhibitory marker PD-L1 and costimulatory marker CD86 regulates the efficiency of effector T cell response.

**Figure 5:**
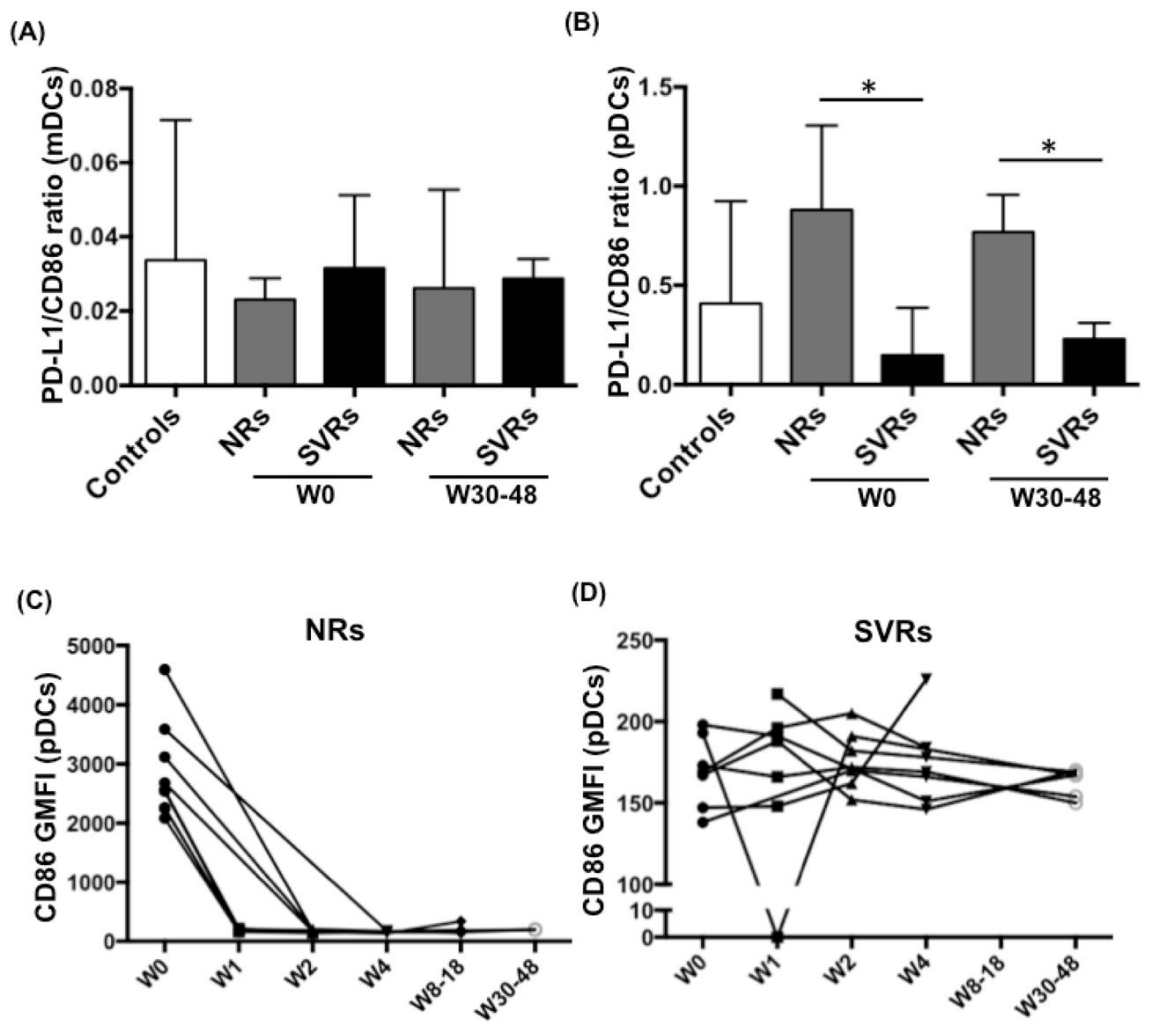
Increased PD-L1/CD86 ratio on pDCs of NRs before and after IFN/RBV treatment PBMCs isolated from seronegative controls, NRs and SVRs were stained with polychromatic antibody cocktail described in supplementary Figure 1. (A) PD-L1/CD86 ratio on mDCs (calculated by dividing the percentage of mDCs expressing PD-L1 by the percentage of mDCs expressing CD86) of seronegative controls, NRs and SVRs before and after IFN/RBV treatment, (B) PD-L1/CD86 ratio on pDCs of seronegative controls, NRs and SVRs before and after IFN/RBV treatment. P values were calculated using Student’s t test (* represents P<0.05). Error bars represent standard deviation. (C) Dot plot representing the CD86 GMFI on pDCs of each NR along the course of therapy. (D) Dot plot representing the CD86 GMFI on pDCs of each SVR along the course of therapy.

To test this hypothesis, we examined CD86 and PD-L1 expression on both DC subsets. As shown in Figure 5A, PD-L1/CD86 ratio of mDCs did not vary significantly between controls and infected individuals. However, the PD-L1/CD86 ratio of pDCs was significantly higher in NRs compared to SVRs (Figure 5B) both before (week 0) and after treatment (week 30–48). We also monitored the expression (GMFI) of CD86 on both DC subsets in each NR and SVR individually. We observed that CD86 expression on mDCs was similar between NRs and SVRs (Data not shown). CD86 expression on pDCs, however, showed differences between NRs and SVRs. At week 0, NRs had higher CD86 expression compared to SVRs (Figures 5C and 5D), but it dropped drastically as soon as week 1 of treatment and remained comparable to SVRs thereafter (Figure 5C). CD86 expression in SVRs remained unchanged (Figure 5D). A recent study in HIV-1/HCV co-infected individuals (the cohort included individuals infected with HCV genotypes 1,2,3 and 4) showed that lower pre-treatment CD86 expression on pDCs, which increases significantly upon treatment, is associated with SVR [34]. Our observation of lower CD86 expression in SVRs compared to NRs partly corroborates this study, however we did not notice any treatment-induced increase in CD86 expression on pDCs). This suggests that in HIV-1/HCV (possibly all HCV genotypes) coinfected individuals, a high baseline maturation state of pDCs may indicate their exhaustion and poor responsiveness to the treatment. Another study with HCV genotype 1 mono-infected individuals did not report elevated CD86 expression in SVRs [35], suggesting that HIV-1 co-infection could be the possible cause of high baseline maturation state of pDCs. Along with our earlier finding (Figure 4G) that treatment-induced reduction in the frequency of CD62L^+^ pDCs associates with non-response suggests that pDCs have an important role to play in the response to IFN/RBV therapy.

**Figure 6:**
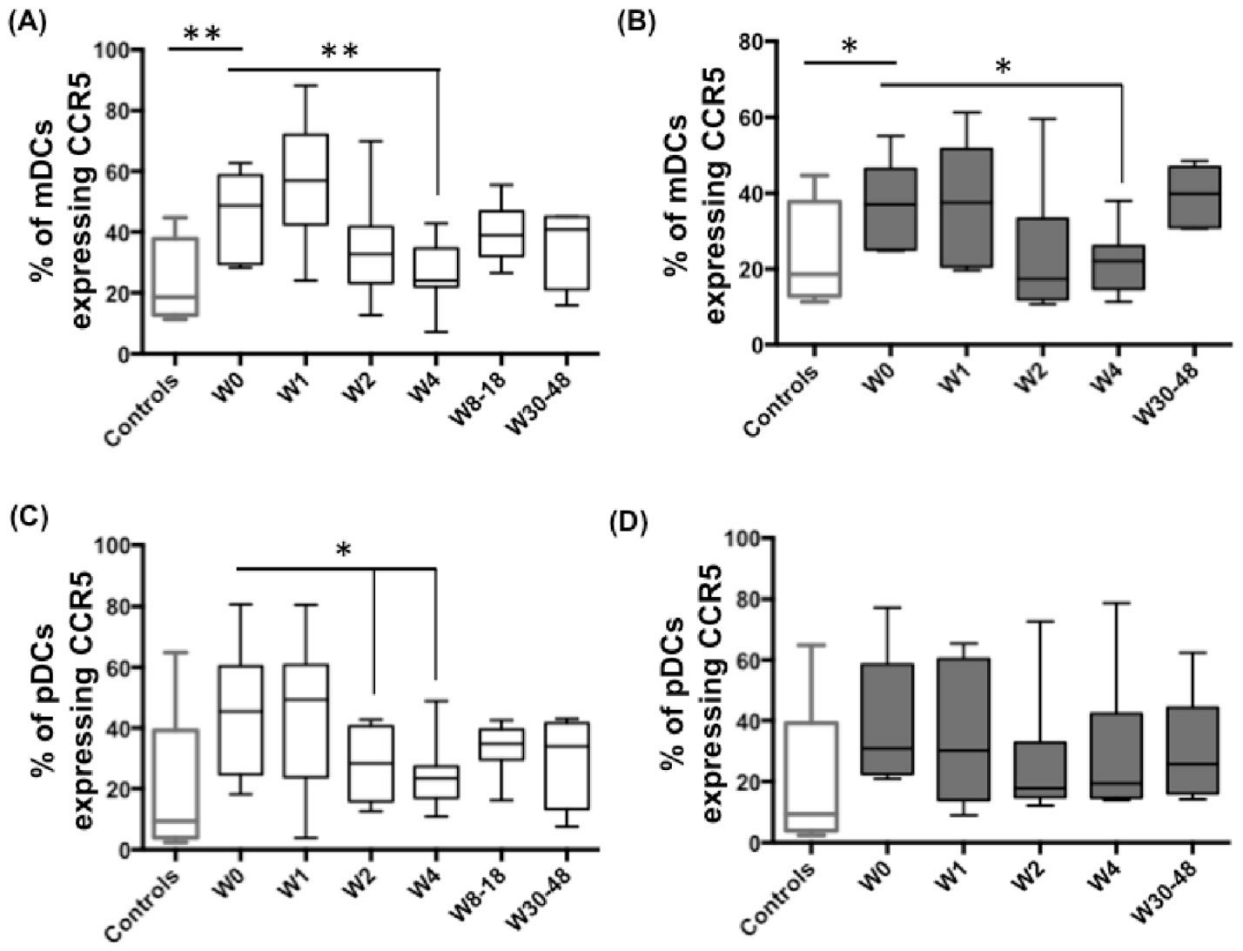
Higher frequency of CCR5+ mDCs in HIV-1/HCV co-infected individuals, but they are downregulated in response to treatment PBMCs isolated from seronegative controls, NRs and SVRs were stained with polychromatic antibody cocktail described in supplementary Figure 1. (A) Box and whiskers graph indicating the percentage of mDCs expressing CCR5 in NRs, and (B) SVRs prior to and during the course of IFN/RBV treatment. (C) Box and whiskers graph indicating the percentage of pDCs expressing CCR5 in NRs, and (D) SVRs prior to and during the course of IFN/RBV treatment. P values were calculated using Student’s t test (* represents P<0.05; ** represents P<0.01).

Higher frequency of CCR5^+^ mDCs in HIV-1/HCV co-infected individuals, but they are downregulated in response to treatment. HCV infection is associated with increased infiltration of mononuclear inflammatory cells many of which express high levels of CCR5 receptor. Moreover, high levels of CCL3, CCL4 and CCL5 (chemokines that bind to CCR5) are found in the HCV-infected liver. Supporting these studies, we observed higher frequency of CCR5^+^ mDCs in both NRs (Figure 6A) and SVRs (Figure 6B), which dropped by week 4 of treatment. On the other hand, pre-treatment frequency of CCR5^+^ pDCs did not differ significantly between the three groups (Figures 6C and 6D), however upon treatment it dropped in NRs (Figure 6C) but not in SVRs (Figure 6D).

SVRs secrete higher amounts of IFN-γ prior to IFN/RBV treatment. Studies on individuals with acute HCV infection have demonstrated that an early, strong and multispecific CD4 and CD8 T-cell responses are related to the resolution of infection. Individuals in whom HCV infection is resolved, a decrease in viremia coincide with a peak in HCV-specific CD8^+^ T cells secreting IFN-γ, a type II IFN. Other studies suggest the ability of IFN-γ to inhibit HCV replication, directly as well as indirectly by potentiating the antiviral effects of IFN-α. It is well known that DCs play a critical role in activation of antigen-specific CD4^+^ and CD8^+^ T-cells.

**Figure 7:**
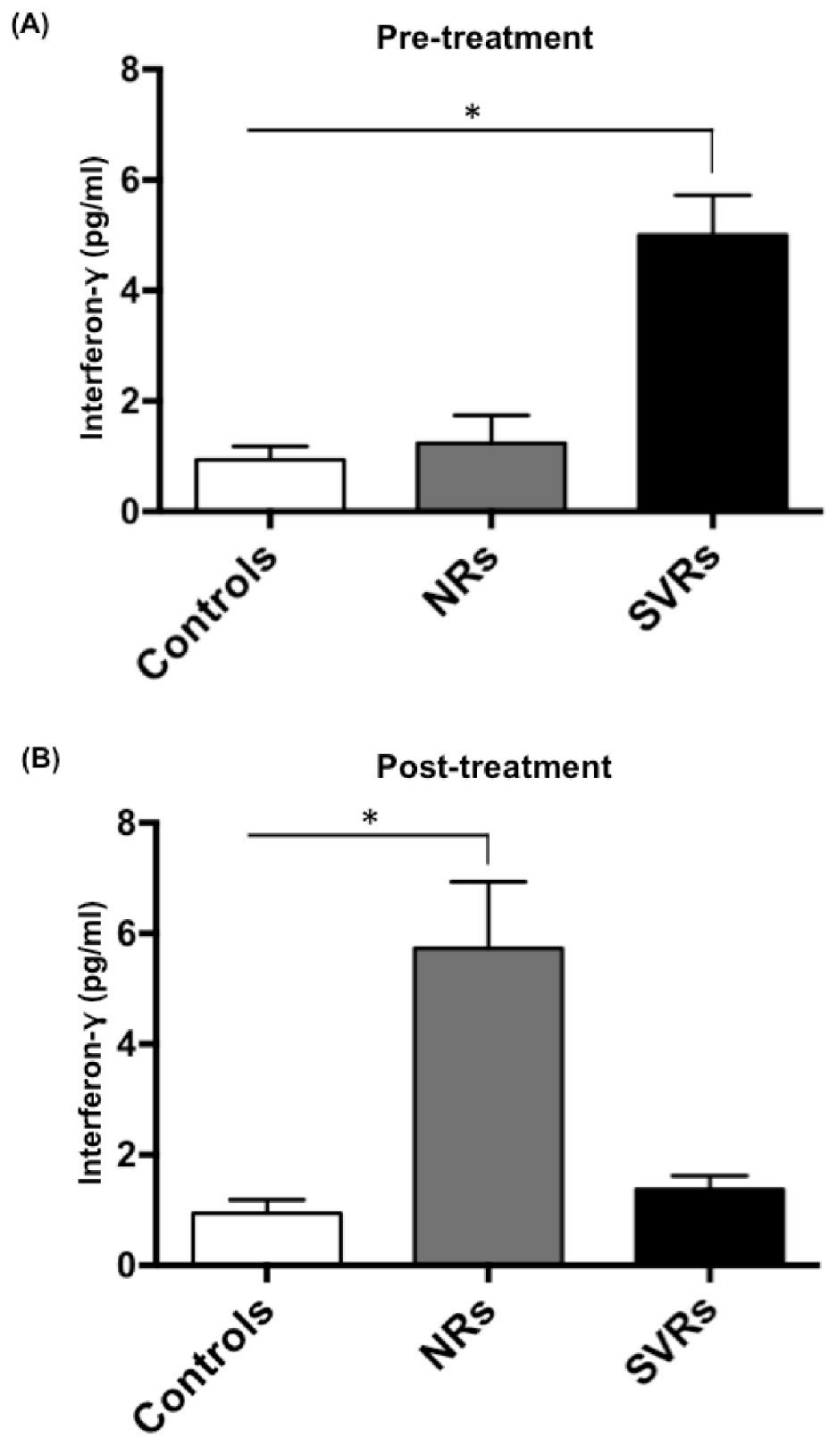
SVRs secrete higher amounts of IFN-γ prior to IFN/RBV treatment PBMCs from seronegative controls, NRs and SVRs were cultured for 24h and levels of various Th1/Th2/Th17 cytokines including IFN-γ were measured using Human Th1/Th2/Th17 Cytokine array (SA Biosciences, Qiagen). (A) Bar graph indicating the concentration of IFN-γ secreted by controls, NRs and SVRs (without any stimulation) at week 0, and (B) week 48 of treatment. P values were calculated using Student’s t test (* represents P<0.05). Error bars represent standard deviation.

Activated CD4^+^/CD8^+^ T-cells are one of the major producers of IFN-γ. DCs can promote natural killer (NK) cell production of IFN-γ as well. We hypothesized that functional dysregulation of DCs can impair the ability of NK cells and T-cells to secrete IFN-γ. Therefore, we compared the levels of IFN-γ secreted by PBMCs of NRs and SVRs both in absence of stimulation (media alone), and stimulation with 1) a cocktail of TLR agonists (mentioned in materials and methods) and 2) IFN-α. The two groups did not show significant differences in IFN-γ secretion when stimulated with TLR agonists and IFN-α (data not shown). However, in absence of stimulation, SVRs secreted higher amounts of IFN-γ compared to controls and NRs at week 0 (Figure 7A). Surprisingly, IFN-γ secretion dropped in SVRs but increased significantly in NRs (Figure 7B). It is important to note that HCV infects PBMCs as well. Therefore, levels of IFN-secreted by patients’ PBMCs could be directly proportional to the percentage of HCV-infected PBMCs (although we have not examined the percentage of HCV-infected PBMCs in our study to validate this statement). Compared to SVRs, NRs have higher viral load at the start of treatment, but still they secrete lower amounts of IFN-γ (Figure 7A). Although IFN/RBV treatment is able to augment the ability of NRs to secrete IFN-γ (Figure 7B), it is unable to clear the virus. In addition to IFN-γ, we also measured the amounts of Th1/Th2/Th17 cytokines (measured using Human Th1/Th2/Th17 Multianalyte ELISA kit) secreted by controls, NRs and SVRs in response to 1) stimulation with TLR cocktail, and 2) stimulation with IFN-α. We did not observe any significant differences (data not shown) between controls and the two groups.

## Materials and Methods

### Acquisition of clinical samples

A total of 23 HIV-1/HCV co-infected (HCV Genotype 1), PEG-IFN-α/RBV treatment naïve individuals were recruited from the Hepatitis Clinic of New York/Weill/Cornell Medical Center in a cohort study. In addition, 6 HIV-1/HCV seronegative, healthy donors (4 males; 2 females) were bought from Biological Specialty (Colmar, PA) as controls. The median age (Interquartile Range), in years, of NRs is 46.5 (43.0–48.0), SVRs is 47.0 (37.0–55.0), and seronegative controls is 51 (48–54). The median CD4 T-cell counts (Interquartile Range), represented as cells/mm^3^, of NRs was 477.5 (434.0–506.0), and SVRs was 361.5 (209.0–493.0). The median HCV serum RNA (Interquartile Range), represented as log10 IU/ml, of NRs is 5.3 (4.7–5.5) and SVRs is 6.6 (6.1–6.8). All enrolled individuals except four NRs: PID 134 (2474 copies/ml), PID 333 (13983 copies/ml), PID 748 (8551 copies/ml) and PID 926 (106518 copies/ml) had HIV viral load less than 400 copies/ml. The study design was approved by the institutional review board of Weill Medical College, Cornell University and conforms to the 1975 Helsinki guidelines for the conduct of human research. Inclusion criteria required individuals to have detectable HCV RNA, be on a stable course of antiretroviral therapy or no antiretroviral agents for at least four weeks prior to initiation of IFN/RBV treatment, and to have a CD4^+^ T-cell count ≥100 cells/mm^3^. Individuals were excluded if they had severe depression, immunodeficiency-related opportunistic infections, on active substance abuse, or were pregnant or lactating.

Subjects were subcutaneously injected with 1.5 μg/kg of PEG-INTRON (Schering Plough, Kenilworth, NJ) once weekly and a 1000–1200 mg daily dose of RBV (Rebitrol, Schering Plough), taken orally for up to 48 weeks. Individuals with HCV RNA below detection (<29 IU/ml) for 24 weeks after treatment termination were considered SVRs. Patient blood was drawn at multiple time points (Week 0, 1, 2, 4, 8, 12, 24, 48 of treatment as well as 24 weeks post-treatment). It is important to note that for few patients (Patient IDs 300, 746, 821, 933) it was not possible to draw blood at week 0 (first day of treatment just prior to the first dose of IFN/RBV). In these patients, blood drawn few days prior to week 0 was used to assess the pre-treatment frequencies of mDCs and pDCs. Therefore, ‘Pre-treatment’ frequencies described in Figure 2A and 2B include patients whose blood was drawn at week 0 as well as patients whose blood was drawn few days prior to week 0. Figures, which specifically indicate ‘week 0’, include only those patients whose blood was drawn at week 0. Week 8–18 SVR samples were not available since they were used up in another study investigating ribavirin pharmacokinetics, HCV RNA and alanine aminotransferase kinetics [36].

### Isolation of peripheral blood mononuclear cells

Peripheral blood drawn from HIV-1/HCV co-infected individuals and seronegative controls was diluted 1:1 with HBSS and used for isolating PBMCs by density gradient centrifugation (400 g; 40 minutes) using Ficoll Paque Plus (GE Healthcare Life Sciences, Piscataway, NJ). After isolation, PBMCs were washed with PBS and cryopreserved using freezing media, 10% DMSO in heat inactivated FBS (Thermo Fisher Scientific, Waltham, MA). At the time of experiment, cells were quickly thawed at 37°C, washed with RPMI-1640 (Mediatech, Herndon, VA) and then with PBS (containing 2 mM EDTA) and immediately used for phenotyping/other experiments.

### Immunofluorescent staining of PBMCs with polychromatic flow cytometry antibody cocktail

We developed a thirteen-color flow cytometry cocktail comprising of cell viability dye (Aqua Blue; Invitrogen, Carlsbad, CA) and twelve antibodies against various DC markers. We have previously utilized a similar cocktail in a cohort of HTLV-1-infected asymptomatic carriers and diseased individuals. Details of the cocktail and gating strategy used to phenotype DCs are mentioned in supplementary figure 1. Briefly, FITC-conjugated Lin-1 (commercially available cocktail containing FITC-CD3, FITC-CD14, FITC-CD16, FITC-CD19, FITC-CD20, and FITC-CD56) was used to gate out non-DCs (Lin-1^+^ cells). Lin-1-cells were used to gate on mDCs (CD11c^+^CD123^−^) and pDCs (CD11c-CD123^+^). Remaining Abs was used to stain for co-stimulation, maturation, activation, and adhesion markers on both DC subsets. Fluorescence-minus-one (FMO) controls were used to set the gates. For staining, PBMCs were suspended in 100 μl staining buffer (PBS supplemented with 2% FBS and 2mM EDTA) and incubated with the Ab cocktail for one hour at 4°C. After incubation, cells were washed with staining buffer, fixed with 1% paraformaldehyde and resuspended in PBS. Thereafter the cells were acquired on a flow cytometer (BD Biosciences FACSAria).

### *In vitro* stimulation of PBMCs with TLR agonists

Cell culture media used for culturing PBMCs consisted of RPMI-1640 supplemented with penicillin (Mediatech, 100 U/ml), streptomycin (Mediatech, 100 μg/ml), HEPES buffer (Mediatech, 10 mM), and 10% heat inactivated FBS. PBMCs (2x105 cells in 300 μl culture media in a U-bottom 96-well plate) were rested for 45 minutes at 37°C with 5% CO_2_ and 90% relative humidity followed by no stimulation or stimulation with (a) cocktail of TLR1/2 (Pam3CSK4 x 3HCl; working concentration of 1 μg/ml), TLR3 (Poly I:C; 10 μg/ml), TLR4 (LPS; 10 μg/ml), TLR6 (Flagellin; 10 μg/ml) TLR7 (Imiquimod; 10 μg/ml), TLR8 (ssRNA40; 10 μg/ml) and TLR9 (ODN2006; 5 μM) agonists and (b) IFN-α (500 IU/ml) for 24 hours. After stimulation, cells were centrifuged at 300 g for 10 minutes and culture supernatant was stored at −80°C for later quantitation of Th1/Th2/Th17 cytokines using ELISA.

### Quantitation of Th1/Th2/Th17 cytokines using ELISA

Culture supernatants collected from TLR agonists- and IFN-treated PBMCs were used to measure the concentration of a panel of 12 cytokines (IL2, IL4, IL5, IL6, IL10, IL12, IL13, IL17A, IFN-γ, TNF-α, G-CSF, and TGF-β1) using Human Th1/Th2/Th17 cytokines multi-analyte ELISA array Kit (SA Biosciences, Qiagen) according to manufacturer’s protocol. Briefly, antigen standards (500 ng/ml) corresponding to each of the twelve cytokines were prepared. 50 μl of assay buffer (supplied with the kit) was pipetted into each well of the 96 well plate (every well has a capture antibody specific to a cytokine) followed by addition of 50 μl antigen standards or undiluted culture supernatant in appropriate wells. Plate was gently shaken and incubated for 2 hours at room temperature. After 2 hours, plates were decanted and washed thrice with washing buffer (supplied with the kit) followed by incubation with biotin-conjugated detection antibodies for 1 hour at room temperature and washed thrice thereafter. In the end, cytokines were detected colorimetrically by addition of avidin-horseradish peroxidase solution followed by addition of enzyme substrate.

### Genotyping of HIV-1/HCV individuals for SNPs rs12979860, rs4803217 and ss469415590 in IFNL genes

Genomic DNA was isolated from PBMCs using SV Wizard genomic DNA isolation kit (Promega) and used to genotype SNPs rs12979860, rs4803217 and ss469415590 by custom designed TaqMan qPCR based allelic discrimination assays. Following primer pair and probes were used for genotyping rs4803217: Forward primer-5′-GCCAGTCATGCAACCTGAGATTTTA-3′, Reverse primer-5′-AAATACATAAATAGCGACTGGGTGACA-3′, Probe for IFNL3-T-5′-FAM-TTAGCCACTTGTCTTAAT-NFQMGB-3′, and Probe for IFNL3-G-5′-VIC-TAGCCACTTGGCTTAAT-NFQMGB-3′; rs12979860: Forward primer-5′-GTGCCTGTCGTGTACTGAACCA-3′, Reverse primer-5′-AGCGCGGAGTGCAATTCA-3′, Probe for IFNL3-C-5′-FAM-CCTGGTTCGCGCCTT-NFQMGB-3′, Probe for IFNL3-T: 5′-VIC-CCTGGTTCACGCCT-NFQMGB-3′; ss469415590: Forward primer-5′-GCCTGCTGCAGAAGCAGAGAT-3′, Reverse primer-5′-GCTCCAGCGAGCGGTAGTG-3′, Probe for IFNL3-ΔG-5′-FAM-ATCGCAGCGGCCC-NFQMGB-3′, and Probe for IFNL3-TT-5′-VIC-ATCGCAGAAGGCC-NFQMGB-3′(where ‘FAM’ is 5-carboxyfluorescein, ‘NFQMGB’ is a nonfluorescent quencher minor groove binder, and ‘VIC’ is 6-carboxyrhodamine). Amount of genomic DNA used per qPCR reaction ranged from 1–3 ng.

### Statistical analysis

Statistical significance between controls, NRs and SVRs was determined using Student’s two-tailed t-test. Data is represented as box and whiskers graph or bar graphs (representing mean ± standard deviation). Prism 6 (GraphPad Software) was used for data analyses and generation of graphs.

## Discussion

Because of significant decline in HIV-related morbidity since the introduction of HAART, liver disease caused by chronic HCV infection has emerged as a major problem in HIV-1/HCV co-infected individuals. HIV co-infection has a negative impact on HCV pathogenesis, and therefore it is recommended to control HIV-1 levels prior to beginning HCV treatment in co-infected individuals. It is also important to note that despite successful response to HAART, many individuals fail to achieve full functional restoration of their immune system. If such individuals get infected with HCV, their ability to mount a successful immune response against HCV could be compromised. HIV-1/HCV co-infection should thus be considered a pathological state with both unique and common features of HIV-1 and HCV mono-infection. Although our immune response can clear HCV [37], virus exposure often proceeds to chronicity. PEG-IFN and RBV combination therapy has been the mainstay of HCV treatment. IFN is a potent antiviral cytokine but is very unpleasant for the patient because of significant side effects such as depression, anemia, thrombocytopenia, neutropenia, diarrhea, and flulike symptoms such as fever, chills, fatigue, headache, and muscle aches. Besides, in individuals infected with the difficult-to-treat HCV genotypes (genotypes 1 and 4), it is curative only 40% 50% of the time. Although first-generation direct-acting antiviral drugs are now being introduced into the clinic; high cost and emergence of drug resistant HCV variants remain important concerns. IFN/RBV will thus be an essential part of HCV treatment and therefore a thorough understanding of its mechanism of action is necessary in order to identify host factors that predictive IFN/RBV treatment response.

Genome-wide association studies have identified SNPs near IFNL3 locus that can predict both spontaneous HCV clearance [26,38] and successful clinical outcome to HCV therapy [38–41]. These SNPs associate with altered mRNA expression of IFNL3, an antiviral cytokine, suggesting that IFNL3 expression levels are associated with HCV clearance and response to therapy [39,40,42]. Interestingly, SNPs in the IFNL3 locus have been shown to affect the expression of IFNL3 not only in liver which is the site of infection but in PBMCs and whole blood [39,40,42–44] with the minor (unfavorable) allele resulting in less IFNL3 expression. Interestingly, unfavorable IFNL3 genotype has been shown to influence DC and NK cells [45,46].

Recent studies have led to identification of two functional SNPs rs4803217 and ss469415590 that are associated with both natural and treatment-based clearance of HCV [27–29,47,48]. In our study, we did not observe any correlation between rs12979860, rs4803217 and ss469415590 genotypes and DC frequencies/phenotype. Given the small size of our cohort, it is necessary to validate our findings in a larger cohort. It will be interesting to study how different IFNL3 polymorphisms affect IFNL3 expression and resulting induction of Interferon-stimulated genes (ISGs) in DCs. It is possible that IFNL3 polymorphisms are responsible for intrinsic defects in DCs and thus responsible for generation of attenuated antiviral immune response.

The importance of DCs in resolving viral infection has been shown for several viruses including HIV-1 and HCV. Individuals that can control HIV-1 and HCV infection are characterized by strong multi epitope-specific CD4^+^ and CD8^+^ T cell response probably reflecting the efficient antigen presentation and T cell activation potential of DCs [49–52]. However, chances of viral clearance after HCV and HIV-1 infection are low, owing to the remarkable ability of these viruses to impair DC functions. At the primary level, viruses can modulate the frequency of various DC subsets by causing their aberrant trafficking, inducing apoptosis, or interfering with their development. Compared to uninfected individuals, HIV-infected [18–22,53] and HCV-infected [12,22–24,54,55] individuals have lower frequency of mDCs and pDCs in blood. During HIV-1 infection, pDCs migrate to the inflamed lymph nodes, where they become activated, apoptotic, and frequently infected with the virus [56,57]. During HCV infection, blood DCs are enriched in liver [24,58–60] suggesting that increased migration of DCs to liver, the primary site of HCV replication, could be the cause of observed drop in DC numbers in blood. Other possibilities are that HCV targets DC precursors [61] or differentiated DCs. In this regard, it has been shown that HCV core, NS3, and NS5 proteins induce apoptosis in mature DCs [62]. Our results show that the frequency of mDCs was diminished in NRs (Figure 2A) whereas the frequency of pDCs was diminished in both NRs and SVRs (although to a greater extent in NRs) (Figure 2B). It is worth noting that HCV-infected individuals with high intrahepatic expression of ISGs prior to treatment achieve the lowest therapy response rates [42,63,64]. Since HCV has evolved a potent strategy to preclude IFN production by infected hepatocytes, the cellular source of IFN responsible for inducing these ISGs in NRs has been unknown for a long time. Recent studies have solved this conundrum by demonstrating the ability of HCV-infected cells to release HCV RNA containing exosomes, which upon recognition by hepatic pDCs trigger IFN production [65]. Therefore, it may be possible that in NRs, the increased expression of ISGs is due to greater frequency/IFN production of pDCs within liver. In fact our results show that prior to treatment, NRs had higher frequency of CCR5^+^ pDCs (Figures 6B and 6C), which could be responsible for their increased migration to liver, the site of inflammation. Upon IFN/RBV treatment, the mDC frequency increased in NRs (Figure 2C) but remained unchanged in SVRs (Figure 2D). On the other hand, the frequency of pDCs reduced even further in NRs (Figure 2E) but remained unchanged in SVRs (Figure 2F). HIV-1 is known to infect mDCs [66]; therefore we were interested in investigating whether mDCs in NRs serve as HIV-1 reservoir and thus become functionally compromised. We did not detect any differences in the frequency of CD4^+^CCR5^+^ mDCs between NRs and SVRs (data not shown).

In addition to direct antiviral effects, IFN-α is recognized to play an important role in regulation of innate and adaptive immune responses. Immunomodulatory effects of IFN-α are partly mediated through their ability to regulate maturation, migratory potential and immunostimulatory capacity of DCs [67–70]. Differential IFN signaling in DCs of NRs and SVRs could underlie intrinsic differences in modulation of DC functions during treatment. Many studies on HCV-infected individuals have shown that various phenotypic and functional characteristics of DCs associate with the treatment outcome. High CD83 expression and low IL-10 production of DCs [8,71] as well as lower chemotaxis index of pDCs to CXCL12 and CXCL10 [35,72] prior to therapy are all associated with SVR. Increased frequency of CD86^+^ mDCs and pDCs upon treatment is also associated with SVR [33]. All these studies suggest that there is a strong link between DC functionality (before or during treatment) and treatment outcome in HCV mono-infected individuals. But not many studies have investigated the same in HIV-1/HCV co-infected individuals. We show that the frequency of mDCs increased during treatment in NRs. This is interesting given the fact that the frequency of CCR7^+^ mDCs was reduced in NRs (Figure 3A). Reduced frequency of CCR7^+^ mDCs could lead to their reduced migration to lymph node resulting in their accumulation in blood. Frequencies of CD54^+^ and CD62L^+^ mDCs were reduced in both NRs and SVRs (Figure 4) indicating that HIV-1/HCV co-infection impairs the maturation potential of mDCs, which fails to recover even after treatment. Also, NRs exhibited reduced frequency of CCR7^+^ pDCs. Our cocktail also included HLA-ABC and HLA-DR but we did not observe any differences in HLA-ABC and HLA-DR expression (data not shown).

Loss of T cell polyfunctionality T is one of the major events during the establishment of persistent viral infection [10,73]. Binding of PD-L1 (on DCs) to PD-1 (on T cells) delivers a coinhibitory signal to T cells leading to their reduced proliferation and effector functions. Our study shows that NRs to IFN/RBV treatment have higher pDC PD-L1/ CD86 ratio (Figure 5), which could be the cause of failed treatment response. Many viruses are known to attenuate the antiviral immune response by upregulating PD-1/PD-L1 expression. PD-1/PD-L1 blockade has shown tremendous promise in restoring the antiviral immune response against many different viruses. In future, it will be important to learn about the exact mechanisms of PD-L1/PD-1 upregulation during chronic viral infections. We also show that prior to treatment, PBMCs from SVRs secrete higher amounts of IFN-γ compared to controls and NRs (Figure 7). IFN-γ, a Th1 cytokine plays an important immunoregulatory role by enhancing the cytotoxic activity of T cells, macrophages and NK cells. Interestingly, IFN-γ levels secreted by PBMCs drop in SVRs at the end of treatment suggesting that infection of PBMCs in SVRs (and not NRs) could be the cause of high pre-treatment levels of IFN-γ and thus could be an important predictor of treatment success. Overall, this study emphasizes the role of DCs in mediating IFN/RBV treatment induced clearance of HCV. It also supports the possibility of using DC-based immunotherapeutic strategies (such as PD-L1 blockade) against HCV.

## Supplementary Material

Supplementary Figure
